# Pneumonia Deterioration Occurring After C-section in a Preeclamptic Patient: A Case Report

**DOI:** 10.7759/cureus.36147

**Published:** 2023-03-14

**Authors:** Dhanista HS. Putra, Kevin Winston, Renaldy Renaldy, Ben B Irwandi, Ali Sakti, Edwin H Martua, Wisnu S Wardhana, Lazuardi G Ilhami, Ikhwan Rinaldi

**Affiliations:** 1 Hospital Medicine, Bhakti Medicare Central Hospital, Sukabumi, IDN; 2 Hospital Medicine, Bhakti Medicare Hospital, Sukabumi, IDN; 3 General Medicine, Faculty of Medicine, Universitas Indonesia, Jakarta, IDN; 4 Obstetrics and Gynecology, Bhakti Medicare Hospital, Sukabumi, IDN; 5 Pulmonology, Bhakti Medicare Hospital, Sukabumi, IDN; 6 Cardiology, Bhakti Medicare Hospital, Sukabumi, IDN; 7 Anesthesiology, Bhakti Medicare Hospital, Sukabumi, IDN; 8 Hematology and Medical Oncology, Cipto Mangunkusumo National General Hospital, Jakarta, IDN

**Keywords:** infection, c-section, pregnancy, preeclampsia, pneumonia

## Abstract

Pneumonia is a type of lung infection that can be caused by bacteria, viruses, or fungi. It is a serious condition that can affect people of all ages, but it is more dangerous for certain populations, such as the elderly, young children, and people with weakened immune systems. Pneumonia can cause higher risk in patients undergoing surgery, including C-sections. In this case report, we present a pregnant woman who was scheduled for a C-section due to preeclampsia and was initially suspected of concomitant pneumonia. The patient successfully underwent the C-section, but unfortunately experienced a deterioration in her pneumonia after the surgery. She was later admitted to ICU and placed on mechanical ventilation due to the deterioration. Despite the acknowledged risks, including the possibility of death, the patient's family chose to bring the patient home due to their belief that there had been no improvement in the patient's condition and a feeling of resignation. In conclusion, pregnant patients who have pneumonia may require an emergency C-section due to several conditions such as preeclampsia, and the C-section can be conducted successfully. However, it is important for physicians to be aware of the potential for the worsening of pneumonia postoperatively. Post-operative pneumonia is a serious condition that can have a significant impact on the health of patients who have undergone a C-section.

## Introduction

Pneumonia is a serious lung infection that can affect all patients, including pregnant women. During pregnancy, the body undergoes many changes such as progesterone and estrogen elevations that can make women more susceptible to infections [1]. The symptoms of pneumonia in pregnancy are similar to those experienced by non-pregnant women. Common symptoms include cough, chest pain, fever, and difficulty breathing [2]. However, pregnant women may also experience additional symptoms such as fatigue, weakness, and loss of appetite [3,4].

Pneumonia can be a serious concern for pregnant women, as it can lead to significant complications for both the mother and the baby [5]. In severe cases, pneumonia during pregnancy can result in premature delivery, low birth weight, or even stillbirth [1,6]. A C-section is a surgical procedure in which a baby is delivered through an incision made in the mother's abdominal and uterine walls. Indications of C-sections include but are not limited to abnormal fetal positioning, previous C-section, placenta previa, preeclampsia, and other maternal health issues [7,8]. In some cases, pneumonia in pregnancy may be so severe that a cesarean section is necessary due to the mortality risk from delivery in pregnant women with severe pneumonia.

In this case report, we present a pregnant woman with suspected pneumonia who was scheduled for a C-section due to preeclampsia. The patient successfully underwent the C-section, but unfortunately experienced pneumonia after the surgery. The purpose of this report is to raise awareness of the potential challenges associated with C-sections in the presence of preeclampsia and suspected pneumonia.

## Case presentation

A 24-year-old pregnant woman with 38-39 weeks of pregnancy G1P0A0 presented to the emergency department on October 15, 2022 with a chief complaint of contractions 12 hours prior to presentation. Other symptoms from the patient included bilateral lower extremity edema. She denied any symptoms of fever, angina, dyspnea, cough, flu, and changes in bowels habit. The patient denied any contact with a positive COVID-19 patient, history of diabetes, heart failure, kidney disease, and liver disease. No similar symptoms were observed in her family. The patient was previously told that she was suspected of having preeclampsia.

The patient was previously diagnosed with pregnancy-related hypertension, which was made around two months prior. She also had previously sought care at a public health center due to swelling in both her extremities. At the public health center, the possibility of preeclampsia was raised and she was referred to a hospital, but unfortunately, she did not follow up on this recommendation.

Physical examination on October 15, 2022 showed abnormal vital signs with blood pressure 160/90 mmHg, respiratory rate 22x per minute, heart rate of 90 beats per minute, and temperature of 36.3 degree Celsius. Additionally, peripheral oxygen saturation was 99%. The patient was alert and oriented, with no signs of altered mental status. Upon lung auscultation, decreased breath sounds were heard in the lower right and left lung fields, though there was no evidence of rales or wheezing. The cardiac exam conducted at the emergency ward showed distant heart sounds. Meanwhile, the abdominal exam revealed the presence of contractions, and mild bilateral peripheral edema was observed in the lower extremities.

On the same day, a complete blood count and additional laboratory tests were conducted on the patient (Table 1). The results showed leucocytosis, with a leucocyte count of 13.5 x 10^3^/µL. The aspartate transaminase (AST) and alanine transaminase (ALT) levels were within normal limits. The rapid COVID-19 antigen test was negative, and all other laboratory tests came back normal. A urine test was also conducted, and it revealed elevated urinary protein with positive 4 (Table 2).

**Table 1:** Laboratory Test on October 15, 2022

Laboratory Test	Value	Reference Value
Hemoglobin (g/dL)	11.1	11.7-15.5
Hematocrit (%)	33	35-47
Leucocyte (10^3^/µL)	13.5	4.5-11.0
Thrombocyte (10^3^/µL)	202	150-440
Erythrocyte Sedimentation Rate (mm/hour)	4.0	0-20
Bleeding time (minute)	3	1-5
Clotting time (minute)	9	3-15
AST (µ/L)	25	<31
ALT (µ/L)	20	<31
Glucose (mg/dL)	76	<200
Hepatitis B antigen	Non-reactive	Non-reactive
Rapid Covid- 19 Antigen Test	Negative	Negative

**Table 2:** Urine test on October 15, 2022

Laboratory Test	Value	Reference Value
Color	Dark Yellow	Yellow
Urine clarity	Cloudy	Clear
Specific gravity	1020	1,005-1,020
pH	5	6-7
Protein	+4	Negative
Glucose	Negative	Negative
Leucocyte	Negative	3-5
Blood	Negative	Negative
Bilirubin	Negative	Negative
Urobilinogen	+1	Negative
Nitrite	Negative	Negative
Ketone	Negative	Negative
Microscopic Examination		
Leucocyte	3-5	3-5
Erythrocyte	1-2	0-2
Epithelial cells	Negative	Negative
Urinary casts	Negative	Negative
Crystal	Negative	Negative
Bacterial	Negative	Negative

**Figure 1:**
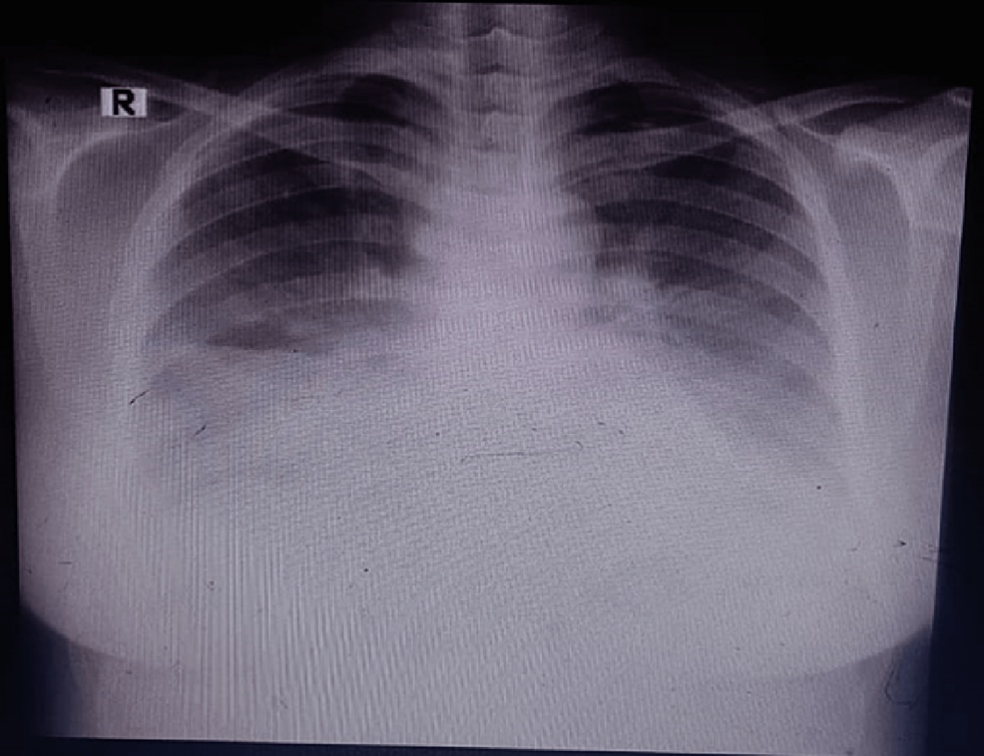
Initial chest x-ray on October 15, 2022, which showed pleural effusion and pericardial effusion.

Her initial chest radiograph (CXR) on October 15, 2022 showed bilateral pleural effusion, suspected cardiomegaly or pericardial effusion, and suspected mild bilateral lung infiltrate (Figure 1). The suspected cardiomegaly and pericardial effusion could not be confirmed at that time due to the masking by the lung infiltrates. Chronic heart failure was unlikely due to the young age of the patient. Pulmonary embolism was also unlikely due to lack of immobility history, lack of sign/symptoms of DVT, lack of hemotypsis and malignancy. Acute respiratory distress syndrome did not happen during this stage due to lack of dyspnea. Thus, at this stage, the patient most likely had mild pneumonia.

Consultation was made to obstetrics and gynecology (O&G) and suspicion of severe preeclampsia was made. Furthermore, based on the x-ray, the patient was suspected of having pneumonia not related to COVID-19. Initial treatments of the patient were ceftriaxone 1 g every 12 hours, ¼ tablet of misoprostol applied to the posterior fornix, magnesium sulfate injection, nifedipine three times a day at a dose of 10 mg, and methyldopa one time a day at a dose of 250 mg.

Due to the presence of severe preeclampsia, the patient was indicated to undergo a C-section procedure. On October 16, 2022, consultations were made with internal medicine and anaesthesiology to assess the patient's tolerance for the surgical procedure. After evaluating the patient, it was determined that the patient was fit to undergo the C-section, which was successfully completed. Post-operation, a complete blood count and other laboratory tests were conducted on October 16, 2022 (Table 3).

**Table 3:** Laboratory test on October 16 and 17, 2022 NA: Not available

Laboratory Test	October 16, 2022	October 17, 2022	Reference value
	Value	Value	
Hemoglobin (g/dL)	11.8	10.8	11.7-15.5
Hematocrit (%)	34	31	35-47
Leucocyte (10^3^/µL)	17.1	18.6	4.5-11.0
Thrombocyte (10^3^/µL)	237	251	150-440
Erythrocyte Sedimentation Rate (mm/hour)	4.0	4.0	0-20
AST (µ/L)	NA	62	<31
ALT (µ/L)	NA	36	<31
Ureum (mg/dL)	NA	34	15-40
Creatinine (mg/dL)	NA	0.5	0.6-1.1
Glucose (mg/dL)	NA	83	<200
Sodium (mmol/L)	NA	132	135-155
Potassium (mmol/L)	NA	3.7	3.4-5.5
Chloride (mmol/L)	NA	100	95-108
Rapid Test COVID-19 antigen	NA	Negative	Negative
Anti-HIV	NA	Non-reactive	Non-reactive

On October 17, the patient reported dyspnea, which started off mild but soon became severe despite oxygen supplementation. The dyspnea was persistent and not influenced by physical activity or changes in body position. The patient also developed symptoms of a cough and fever.

Physical examination on October 17, 2022 showed abnormal vital signs with blood pressure 160/100 mmHg, respiratory rate 40x per minute, heart rate 160 beats per minute, and temperature of 38.3 degree Celsius. Additionally, oxygen saturation deteriorated to 70% room air. There was altered mental status. Lung auscultation revealed decreased vesicular sound on bilateral pulmonary areas. Rales were present during auscultation. Extremity examination showed presence bilateral peripheral edema.

**Figure 2:**
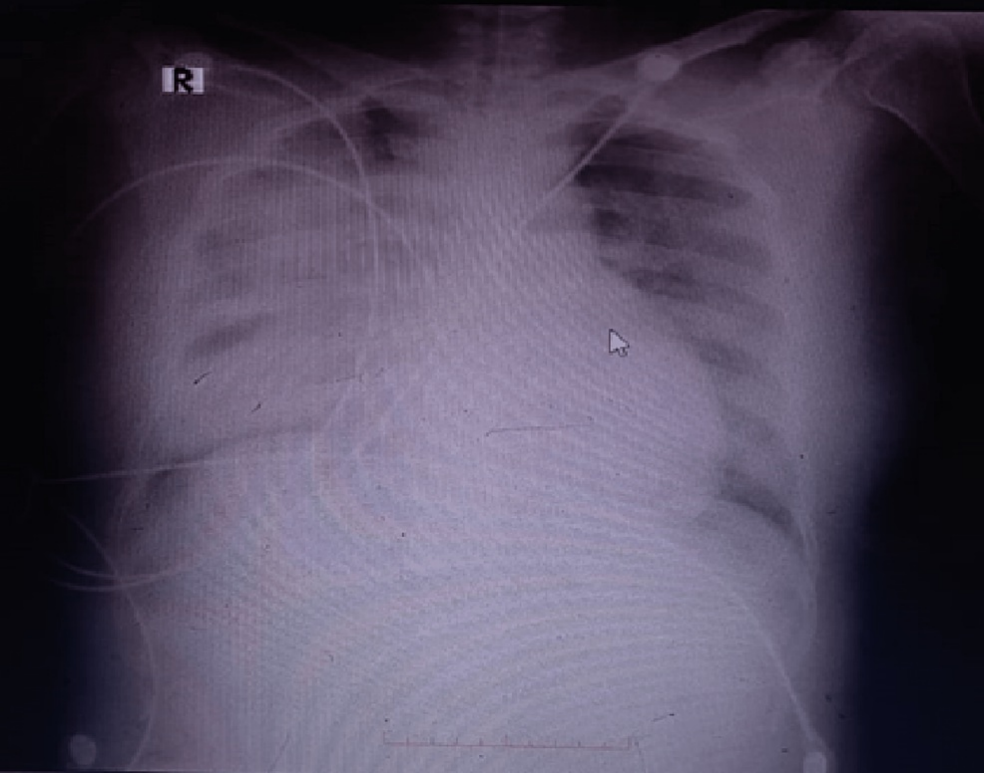
Chest x-ray taken on October 17, 2022

On October 17, 2022, another chest x-ray was performed. Comparison of this x-ray with the one taken on October 15, 2022, showed changes in the volume of pleural and pericardial effusions. Overall, the CXR showed bilateral pleural effusion, consolidation on right lung, hilar infiltrates, and presence of air bronchogram on bilateral lower lobes (Figure 2). The pleural effusion was thought to be caused by the pneumonia and/or severe preeclampsia.

Complete blood count and other laboratory examinations were performed on October 17, 2022 (Table 3). The patient’s lab revealed that she had leukocytosis with 18.6 (10^3^/µL) of leucocyte, elevation of aspartate transaminase (AST) and normal alanine transaminase (ALT). Rapid test COVID-19 antigen was negative and other laboratory tests hyponatremia and hypochloremia. An arterial blood gas analysis was conducted on October 17, 2022, and the results are summarized in Table 4. The analysis showed that the patient was suffering from acute uncompensated respiratory acidosis, which was likely caused by pneumonia. The diagnosis of pneumonia was supported by previous infiltrate on October 15 and recent CXR on October 17. Peripartum cardiomyopathy (PPCM) was also a possibility, but this was disproven but normal echocardiography later on.

**Table 4:** Arterial blood gas results on October 17, 2022

Laboratory Test	Value	Reference Value
pH	7.03	7.35-7.45
HCO_3_ (mmol/L)	37	22-26
sO2 (mmol/L)	55	94-98
tCO_2_ (mmol/L)	18	22-26
Base excess (mmo/L)	-14	-2.5-2.5
pO_2_ (mmHg)	43	83-108
pCO_2_ (mmHg)	63	35-45
Temperature	37	35-37

To further evaluate the patient's condition, the patient was consulted with specialists in internal medicine, pulmonary medicine, and anesthesiology. Based on these consultations, additional treatments were recommended, including oxygen therapy at a flow rate of 15 liters per minute, monitoring of blood gas levels, re-checking of blood laboratory results, and transfer of the patient to the Intensive Care Unit. Furthermore, based on the latest examination data, it was decided to change the patient's treatment plan was adjusted to include meropenem 1 g intravenous, given three times a day, and supportive therapy, including intubation and mechanical ventilation through ventilator. The pulmonologists also recommended acetylcysteine 3x200 mg, and bronchodilator inhalation every eight hours.

Intubation procedure was conducted using midazolam 5 mg, fentanyl 100 microgram and rocuronium 50 mg post-intubation, physical examination still showed abnormal vital signs, including a blood pressure of 148/95 mmHg, a respiratory rate of 16 breaths per minute, a regular heart rate of 175 beats per minute, and a temperature of 36 degrees Celsius. The patient's peripheral oxygen saturation was 100% on a ventilator mode of synchronized intermittent mandatory ventilation (SIMV) with volume control (VC), tidal volume of 450mL, a ratio of inspiration:expiration of 1:2, positive end-expiratory pressure (PEEP) 5 mmHg, FiO_^2^_ of 100%, and a respiratory rate of 16x/minute.

Lung auscultation revealed decreased vesicular sound in the right and left lung fields, with rales present. Cardiac auscultation showed distant heart sounds. Abdominal examination showed abdominal breathing. Examination of the extremities showed the presence of bilateral peripheral edema. The patient's diuresis was 1.6 cc/kg body weight per hour.

On October 18, 2022, production of a red-black color in the nasogastric tube was observed. Thus, a diagnosis of stress ulcer was made. Thus, the patient was given additional bolus of omeprazole. Furthermore, the patient was given additional treatments, including furosemide, given intravenously two times a day, moxifloxacin, given once a day at a dose of 400 mg, tranexamic acid, given three times a day, bronchodilator inhalation, and a repeat complete blood count. The furosemide was recommended by O&G specialist for postpartum management of severe preeclampsia. However, the internal medicine doctor recommended delaying the administration of loop diuretics until the results of an echocardiogram examination were available.

Based on the results of the complete blood count, a working diagnosis of anemia caused by upper gastrointestinal tract bleeding from stress ulcers was established (Table 5). The internist provided additional advice, including a transfusion of one unit of packed red cells and administration of daily drip of omeprazole, given five vials per 24 hours.

**Table 5:** Laboratory examinations on October 18, 2022

Laboratory Test	Value	Reference Value
Hemoglobin (g/dL)	8.3	11.7-15.5
Hematocrit (%)	25	35-47
Leucocyte (10^3^/µL)	18	4.5-11.0
Thrombocyte (10^3^/µL)	216	150-440
Erythrocyte Sedimentation Rate (mm/hour)	3.0	0-20
Blood group	O/Rh +	

On October 19, 2022, the patient's physical examination revealed abnormal vital signs with a blood pressure of 155/108 mmHg on the monitor, a respiratory rate of 27 breaths per minute, a heart rate of 102 beats per minute, and a temperature of 36.2°C. Additionally, the patient's oxygen saturation was 99% on a ventilator mode of SIMV VC, with a tidal volume of 450, an I:E ratio of 1:2, a PEEP of 10 mmHg, a FiO_2_ of 80%, and a frequency of 20. During lung auscultation, a decreased vesicular sound was heard in both the right and left lungs, and rales were present. The cardiac auscultation revealed a distant heart sound. The patient's diuresis was 2.9 cc/kg body weight per hour.

Cardiac echocardiography was also performed the same day and the results showed normal ventricle volumes, systolic function, and diastolic function, as well as normal valves. The tricuspid annular plane systolic excursion (TAPSE) was measured at 2 cm, with an ejection fraction (EF) of 54%, normal heart valves, no evidence of thrombus, and no pericardial effusion.

Based on the laboratory results, it appeared that the patient still had anemia and had developed acute renal failure (Table 6). The low hemoglobin levels suggested that the transfusion of PRC may not have been adequate or there may be ongoing blood loss from an underlying condition such as the stress ulcer. While there was an indication for upper gastrointestinal endoscopy in this patient, no endoscopy was performed since endoscopy modality was not available in our hospital. The elevated creatinine and urea levels suggested impaired kidney function, which could be due to dehydration, sepsis, or the use of nephrotoxic drugs. The latest diagnosis of this date for the patient was sepsis, acute respiratory distress syndrome from pneumonia, stress ulcer, acute kidney injury (AKI), and hypoalbuminemia. The latest CURB-65 pneumonia score was 3, indicating 14.0% 30-day mortality.

**Table 6:** Laboratory examinations on October 19, 2022

Laboratory Test	Value	Reference Value
Ureum (mg/dL)	103	15-40
Creatinine (mg/dL)	3.0	0.6-1.1
GFR (mL/min/1.73 m^2^)	27.3	
Glucose (mg/dL)	73	<200
Albumin	2.7	3.5-5
Natrium (mmol/L)	138	135-155
Kalium (mmol/L)	4.2	3.4-5.5
Chlorida (mmol/L)	110	95-108

On October 20, 2022, the patient's family made the decision to voluntarily discharge the patient from the hospital and bring her home. Informed consent was obtained from the family. Despite the acknowledged risks, including the possibility of death, the patient's family chose to bring the patient home due to their belief that there had been no improvement in the patient's condition and a feeling of resignation.

## Discussion

Pneumonia can have significant effects on a woman's ability to undergo a C-section surgery. Pneumonia is a serious lung infection that causes inflammation and fluid buildup in the lungs, resulting in impaired diffusion. This can be especially concerning in the context of surgery, where a patient needs to be healthy and able to breathe normally in order to undergo the procedure safely.

One of the effects of pneumonia on a C-section is that it may delay or even prevent the surgery from taking place [9]. If a woman has pneumonia, she may need to be hospitalized and treated with antibiotics and supportive care before she is healthy enough to undergo a C-section. This can result in a delay in the delivery of the baby and additional drugs, which can be stressful for both the mother and the baby [5].

Additionally, pneumonia can also increase the risks associated with C-section surgery [10-12]. For example, the respiratory distress caused by pneumonia may lead to difficulty breathing and low oxygen levels, both of which can be dangerous for the mother and the baby during surgery. Moreover, pneumonia can weaken the immune system and make it more difficult for the body to fight off infections, increasing the risk of postoperative infections and other complications [3,4,9].

A difficult issue arises when a patient needs a C-section for an emergency condition, but the patient has pneumonia. In our case, the patient had preeclampsia which was determined to need a C-section, but the presence of pneumonia complicated the case. Very little literature is available describing C-sections in patients with pneumonia. A case report by Lau et al. described an emergency C-section preterm pregnant woman with severe COVID-19 pneumonia and the patient was discharged later on due to improvement [13]. In contrast, our patient deteriorated post-C-section and initially had a suspicion of pneumonia based on CXR without pneumonia symptoms.

Patients who have had a C-section are at an increased risk of developing pneumonia due to several factors [14,15]. The surgical incision can cause decreased lung function, making it harder for patients to clear secretions and increasing the risk of respiratory infections. Additionally, the use of anesthesia and medications during surgery can also weaken the immune system and make patients more susceptible to infections [16,17]. It is speculated that a combination of these factors and preeclampsia before the C-section causes pneumonia deterioration in our patient post-C-section.

Our patient also had AKI post-C-section. Patients who have had a C-section are at an increased risk of developing AKI due to several factors, including decreased blood flow to the kidneys, and dehydration [18,19]. Additionally, patients who have had a C-section may be at a higher risk for blood clots, which can also cause AKI by blocking blood flow to the kidneys. We believe that in our patient, sepsis from pneumonia combined with hypovolemia caused AKI.

Limitation

The limitations of this case report include the absence of initial hepatic function and kidney function tests. Our hospital lacked some necessary tests such as cholinesterase, and the kidney function test was delayed due to the insurance limitations of the patient. Arterial blood gas was not checked during admission since it was thought at the time there was no need for arterial blood gas due to a lack of dyspnoea symptoms. Additionally, the data on lactate and anion gap were unavailable.

## Conclusions

Pregnant patients who have pneumonia may require an emergency C-section due to several conditions such as preeclampsia and the C-section can be conducted successfully. However, it is important for physicians to be aware of the potential for the worsening of pneumonia postoperatively. Post-operative pneumonia is a serious condition that can have a significant impact on the health of patients who have undergone a C-section. Early diagnosis and prompt treatment are crucial to minimize the risk of complications and improve the chances of a positive outcome.
